# Tobacco exposure in adults and children with proteinuric glomerulopathies: a NEPTUNE cohort study

**DOI:** 10.1186/s12882-023-03073-w

**Published:** 2023-02-09

**Authors:** Linda Wang, Bayle Smith-Salzberg, Kevin EC Meyers, Dorey A. Glenn, Katherine R. Tuttle, Vimal K. Derebail, Tammy M. Brady, Keisha Gibson, Abigail R. Smith, Michelle M. O’Shaughnessy, Tarak Srivastava, Gentzon Hall, Jarcy Zee, Markus Bitzer, Christine B. Sethna

**Affiliations:** 1 Cohen Children’s Medical Center of NY, New Hyde Park, NY USA; 2 Children’s Hospital of Philadelphia, Philadelphia, PA USA; 3 Division of Nephrology and Hypertension, UNC Kidney Center, University of North Carolina at Chapel Hill, Chapel Hill, NC USA; 4 Providence Medical Research Center, Providence Health Care, Spokane, WA USA; 5 Nephrology Division and Kidney Research Institute, University of Washington, Seattle, WA USA; 6 Johns Hopkins University School of Medicine, Baltimore, MD USA; 7 Arbor Research Collaborative for Health, Ann Arbor, MI USA; 8 Cork University Hospital, Cork, Ireland; 9 Childrens Mercy, Kansas City, MO USA; 10 Division of Nephrology, Duke University School of Medicine, 269-01 76th Avenue, 11040 Durham, NC USA; 11 Department of Biostatistics, Epidemiology, and Informatics, University of Pennsylvania Perelman School of Medicine, Philadelphia, PA USA; 12 University of Michigan, Ann Arbor, MI USA

**Keywords:** Cigarette smoking, Second-hand smoking, Nephrotic syndrome, Cotinine, Cardiovascular risk factors

## Abstract

**Background:**

Tobacco exposure has been recognized as a risk factor for cardiovascular disease (CVD) and progression of kidney disease. Patients with proteinuric glomerulopathies are at increased risk for cardiovascular morbidity and mortality. Multiple studies have linked tobacco exposure to CVD and chronic kidney disease, but the relationships between smoking and proteinuric glomerulopathies in adults and children have not been previously explored.

**Methods:**

Data from the Nephrotic Syndrome Study Network (NEPTUNE*)*, a multi-center prospective observational study of participants with proteinuric glomerulopathies, was analyzed. 371 adults and 192 children enrolled in NEPTUNE were included in the analysis. Self-reported tobacco exposure was classified as non-smoker, active smoker, former smoker, or exclusive passive smoker. Baseline serum cotinine levels were measured in a sub-cohort of 178 participants.

**Results:**

The prevalence of active smokers, former smokers and exclusive passive smoking among adults at baseline was 14.6%, 29.1% and 4.9%, respectively. Passive smoke exposure was 16.7% among children. Active smoking (reference non-smoking) was significantly associated with greater total cholesterol among adults (β 17.91 95% CI 0.06, 35.76, p = 0.049) while passive smoking (reference non-smoking) was significantly associated with greater proteinuria over time among children (β 1.23 95% CI 0.13, 2.33, p = 0.03). Higher cotinine levels were associated with higher baseline eGFR (r = 0.17, p = 0.03).

**Conclusion:**

Tobacco exposure is associated with greater risk for CVD and worse kidney disease outcomes in adults and children with proteinuric glomerulopathies. Preventive strategies to reduce tobacco exposure may help protect against future cardiovascular and kidney morbidity and mortality in patients with proteinuric glomerulopathies.

**Supplementary Information:**

The online version contains supplementary material available at 10.1186/s12882-023-03073-w.

## Introduction

In the United States, approximately 34.1 million adults smoke cigarettes and 4.5 million middle and high school students currently use tobacco products [1]. Although passive smoke exposure to nonsmokers is declining, 20.8% of adults and 40.6% of U.S. children are still exposed to second-hand smoke [2]. Active and passive smoke exposure are associated with increased cardiovascular disease (CVD) risk in adults [3] and passive smoke exposure is an independent risk factor for CVD development in children. Children with passive smoke exposure were found to have higher systolic blood pressure, daytime blood pressure load and blood pressure variability compared to non-smokers [4].

Tobacco exposure has also been recognized as a risk factor for progression of kidney disease and proteinuria in adults and children [5–7]. Nicotine and its metabolites are known to be directly cytotoxic to glomerular visceral epithelial cells (i.e. podocytes); an essential cellular component of the glomerular filtration barrier. Several studies have demonstrated the direct damage of nicotine to podocytes, which leads to proteinuria and progression of chronic kidney disease (CKD) [8–13].

Patients with primary proteinuric glomerulopathies, which include minimal change disease (MCD), focal segmental glomerulosclerosis (FSGS) and membranous nephropathy (MN) [14, 15], are at increased risk for CVD. As such, CVD is reported to be the leading cause of death in primary nephrotic syndrome [16]. The increased risk for CVD is due in part to alterations in lipoprotein metabolism, hypertension, pro-thrombotic risks and medication exposures. It is also possible that proteinuria is a risk factor for CVD independent of lipid levels. Additionally, adults and children with proteinuric glomerulopathies are at risk for decline in kidney function and kidney failure.

Multiple studies have associated tobacco exposure with increased risk for CVD and CKD. However, relationships between tobacco exposure, CVD, and CKD have not specifically been examined in patients with proteinuric glomerulopathies, who might be at particularly increased risk for adverse cardiovascular and kidney outcomes. The objective of this study was to describe the prevalence of tobacco exposure in adults and children with proteinuric glomerulopathies enrolled in the Nephrotic Syndrome Study Network (NEPTUNE) and to examine associations between tobacco exposure, additional cardiovascular risk factors, and kidney disease outcomes in this population. We hypothesized that active and passive smoke exposure would be associated with hypertension, dyslipidemia, kidney function decline, proteinuria, and the presence of arteriosclerosis by kidney biopsy.

## Methods

### Nephrotic syndrome study network (NEPTUNE)

NEPTUNE is a multi-center, prospective, longitudinal observational cohort study of adults and children with proteinuric glomerulopathies. The design of the NEPTUNE study has been previously described [17]. Participants were recruited at the time of kidney biopsy from 23 participating sites across North America. Criteria for enrollment included a urine protein to creatinine ratio (UPC) of ≥ 0.5 g/g (NEPTUNE 1 cohort) or > 1.5 g/g (NEPTUNE 2 cohort). Those with systemic disease (e.g. systemic lupus erythematosis, diabetes mellitus, systemic vasculitis), history of solid organ transplant, or life expectancy < 6 months were excluded. Participants were classified according to histologic diagnoses of MCD, FSGS, MN or other glomerulopathy. Participants were followed for five years with visits every four months in the first year of enrollment and subsequently every six months. Participants enrolled between July 1, 2010 and June 12, 2019 were included in this analysis. The study protocol was approved by the Institutional Review Board at each participating site and informed consent/assent was obtained from participants.

### Tobacco exposure

The prevalence of tobacco exposure among NEPTUNE participants was the primary outcome of the study. Tobacco exposure was self-reported at each study visit by questionnaire. Participants were classified as: (1) non-smokers, (2) active smokers, (3) former smokers, or (4) exclusive passive smokers; classifications were mutually exclusive. Non-smoker was defined as an individual who does not currently smoke or have previous history of smoking, and without exposure to any household members who smoke. Active smoker was defined as a participant who currently smokes cigarettes, pipes or cigars. Former smokers were defined as an individual who reported previous smoking. Exclusive passive smokers were participants with smoke exposure in the household and no history of current or former smoking.

Serum cotinine (a metabolite of tobacco) levels were collected from baseline blood samples in a nested cohort of 178 adults and children enrolled prior to 2015. All participants classified as active or passive smokers at baseline prior to this date had serum cotinine levels measured. An additional random sample of 98 reported non-smokers were chosen for cotinine sampling using a computer random number generator. Serum cotinine measurements were run in duplicate using ELISA kits (ABNOVA, Taiwan) as per the manufacturer’s protocol at the Children’s Hospital of Philadelphia. Participants with levels > 10 ng/ml were considered to be active smokers. If levels were > 1 ng/ml but < 10 ng/ml, participants were considered to have passive smoke exposure. Participants with levels < 1 ng/ml were classified as nonsmokers [18].

### Cardiovascular risk factors

The cardiovascular risk factors of interest included blood pressure (BP) and lipid measures. Casual BP was measured in triplicate according to established hypertension guidelines [19, 20] at each study visit using a validated oscillometric device and the average of the last two readings was used in analyses. BP status was classified at each study visit. Hypertensive BP status was assigned if their average BP was in the hypertensive range or if a clinical diagnosis of hypertension was documented in their medical record. Hypertensive BP was defined as BP > 130/80 mmHg in adults and children ≥ 13 years of age; and BP > 95th%ile by age, sex, and height in children < 13 years [19, 21]. Elevated BP status was defined as BP 120–129/80 mmHg in adults and children ≥ 13 years of age; and BP 90-95th%ile by age, sex, and height in children < 13 years [19, 21]. The BP index (BP/95th percentile BP) was calculated for children < 18 years as a method of standardizing and comparing blood pressure across various age groups. BP index ≥1 was significant for hypertensive BP. Additionally, participants were classified as having hypertension if there was a diagnosis of hypertension in the medical record or by self-report.

Non-fasting serum samples for lipid studies were measured centrally at the University of Michigan. Dyslipidemia was defined as any of the following: high density lipoprotein (HDL) < 40 mg/dL; non-HDL ≥ 145 mg/dl (< 18 years), ≥ 160 mg/dl (≥ 18 years); or triglycerides ≥ 100 mg/dL (0–9 years), ≥ 130 mg/dL (10–17 years), or ≥ 150 mg/dL (≥ 18 years) [22].

### Cardiovascular disease events and co-morbid conditions

Cardiovascular disease diagnoses including coronary artery disease, stroke, heart failure and peripherical vascular disease were queried by self-report at the baseline visit and all follow-up visits. Additionally, data on thromboembolic events and cancer diagnoses were collected.

### Kidney disease outcomes

Kidney disease outcomes were pre-defined in the NEPTUNE protocol [17]. Outcomes of interest included longitudinal continuous measurements of UPC and estimated glomerular filtration rate (eGFR) as well as dichotomous outcomes of complete remission, CKD progression, and kidney failure. UPC was measured centrally at the University of Michigan. In adults, eGFR was calculated using the Chronic Kidney Disease-Epidemiology (CKD-Epi) 2009 formula while the modified Schwartz formula was used for children under the age of 18 [23, 24]. Complete remission was defined as UPC < 0.3 g/g at any time point after nephrotic syndrome diagnosis. CKD progression was defined as ≥ 40% eGFR decline compared to previous follow up and eGFR < 90 ml/min/1.73m^2^. Kidney failure was defined as eGFR < 15 ml/min/1.73m^2^ or requiring kidney replacement therapy (KRT) or transplant.

### Pathology

Kidney biopsy specimens were scored by core NEPTUNE pathologists. Arteriosclerosis and arterial hyalinosis were graded from 0 to 3+.

### Clinical characteristics

The NEPTUNE database was abstracted for anthropometric measurements including weight, height, and body mass index (BMI). For children, birth weight was recorded and BMI percentiles were calculated by age and sex. Obesity was defined as BMI > 30 kg/m^2^ for adults and BMI percentile > 95 percentile for children [25]. Race, sex, alcohol use, illicit drug use, English as a primary language, maternal education level, and employment status were self-reported or reported by parents of children. Pharmacotherapies recorded included glucocorticoid, calcineurin inhibitor (CNI), and renin-angiotensin-aldosterone system blocker (RAAS) use.

### Statistical analysis

Analyses were stratified by participant age: adult (≥ 18 years) and pediatric (< 18 years). Baseline descriptive characteristics of the self-reported tobacco exposure groups included mean, standard deviation, median and interquartile range (IQR) for continuous variables as well as frequency counts and percentages for categorical variables. To identify the prevalence of tobacco exposure, frequencies and proportions of self-reported non-smokers, active smokers, former smokers and passive smokers were calculated. Comparisons across tobacco exposure groups in adults were done with chi-square test for categorical variables and ANOVA or Kruskal Wallis test for continuous variables, as applicable. Given the small number of active/former smokers in the pediatric cohort, comparisons of passive smokers vs. non-smokers only were made in the pediatric cohort, using chi-square, t-tests and Mann-Whitney U tests, as appropriate.

Longitudinal analyses were performed using generalized estimating equations (GEE) to examine the association of self-reported tobacco exposure with cardiovascular risk factors (BP and lipids) and kidney disease outcomes (UPC, eGFR and complete remission). Outcomes of CKD progression and kidney failure were analyzed using time-varying Cox survival analysis. Given the limited number of cardiovascular disease events and co-morbid conditions, regression analyses were not carried out for these outcomes. Models were adjusted for potentially confounding variables, chosen *a priori* based on known associations with outcomes of interest: age, sex, race, weight status, glomerular diagnosis, UPC, log eGFR, and steroid use. Tobacco exposure, age, weight status, UPC, log eGFR and steroid use were entered as time-varying variables. The UPC model did not include UPC and eGFR model did not include eGFR as confounding variables. The model for kidney failure was not performed in children and was only adjusted for age and sex in adults due to a small number of participants experiencing this outcome. CKD progression in children was also adjusted only for age and sex due to a small number with the outcome. Active smokers and exclusive past smokers were the main groups of interest for regression analyses, using non-smokers as the reference group.

Demographic information of the nested cohort with cotinine data was compared to those without cotinine data using chi-square, t-tests and Mann-Whitney U tests, as appropriate. To compare self-reported tobacco exposure to cotinine classification of tobacco exposure, sensitivity (i.e. the probability that self-reported tobacco use will be positive when the cotinine results are positive) and specificity (i.e. the probability that self-reported tobacco use will be negative when the cotinine results are negative) were calculated. Baseline UPC, eGFR, BP and lipids were examined for correlation with cotinine level using Spearman correlation. SPSS 26.0 (IBM Inc) was used to analyze the data. A two-tailed p-value < 0.05 was the criterion for statistical significance.

## Results

This study included 371 adults and 192 children enrolled in NEPTUNE. Among adult participants at baseline, the mean age was 45.9 ± 18 years, 106 (55.5%) participants were male, and 34 (20.7%) identified as Black race (Table 1). The median duration of follow up was 3.6 (IQR 2.3, 4.5) years. Self-reported tobacco exposure status at baseline was 51.5% (N = 191) non-smokers, 14.6% (N = 54) active smokers, 29.1% (N = 108) former smokers, and 4.9% (N = 18) exclusive passive smokers. Active smokers (missing 7.4%) reported smoking for a median of 20 (IQR 10, 35) years and smoked a median 0.5 (IQR 0.25, 1) packets of cigarettes per day. There were 27 participants that reported pipe or cigar use. The prevalence of passive smoke exposure among all adults (including active and former smokers) was 13.2% (N = 49). Of former smokers who responded (missing 72.2%), the tobacco-free period was a median 0.88 (IQR 0, 9.8) years. Comparisons among the tobacco exposure groups showed that the former smoker group was older than the other groups. The former smoker group had the highest proportion of male participants (70.4%), followed by active smokers (68.5%) and non-smokers (55.5%). The active smoker group had the highest proportion of participants who identified as Black race (41.5%), reported illicit drug usage (22.2%), or were unemployed (50.0%).

**Table 1:** Baseline Demographic and Clinical Characteristics of Adult NEPTUNE Participants by Tobacco Exposure Groups

N (%) or Mean±SD N = 371	Non-smoker N = 191	Active smoker N = 54	Former smoker N = 108	Exclusive Passive smoker N = 18	p-value
Age	43.7±16.3	47.6±13.5	49.5±16.0	41.5±15.0	0.01
Sex: Male	106 (55.5%)	37 (68.5%)	76 (70.4%)	6 (33.3%)	<0.01
Black Race	39 (20.7%)	22 (41.5%)	18 (16.8%)	5 (27.8%)	<0.01
Drug use	9 (4.7%)	12 (22.2%)	18 (16.7%)	1 (5.6%)	<0.01
Alcohol use: None	97 (51.1%)	25 (46.3%)	40 (37.4%)	9 (50.0%)	0.27
Daily	4 (2.1%)	3 (5.6%)	7 (6.5%)	0 (0.0%)	
3-4 times a week	7 (3.7%)	4 (7.4%)	9 (8.4%)	2 (11.1%)	
1-2 times a week	23 (12.1%)	6 (11.1%)	16 (15.0%)	2 (11.1%)	
1-2 times a month	23 (12.1%)	6 (11.1%)	22 (20.6%)	2 (11.1%)	
< once a month	36 (18.9%)	10 (18.5%)	13 (12.1%)	3 (16.7%)	
English as primary language	142 (74.3%)	46 (85.2%)	83 (76.9%)	12 (66.7%)	0.30
Employment Status: Not Employed	45 (23.7%)	27 (50.0%)	44 (40.7%)	7 (38.9%)	<0.01
Employed	127 (66.8%)	26 (48.1%)	62 (57.4%)	10 (55.6%)	
Homemaker	10 (5.3%)	0 (0.0%)	0 (0.0%)	1 (5.6%)	
Not Applicable	8 (4.2%)	1 (1.9%)	2 (1.9%)	0 (0.0%)	
Education Level:					0.49
High School diploma	45 (23.6%)	9 (16.7%)	21 (19.6%)	2 (11.1%)	
2-year Associates degree/certificate	25 (13.1%)	6 (11.1%)	7 (6.5%)	2 (11.1%)	
4-year College degree	25 (13.1%)	6 (11.1%)	12 (11.2%)	1 (5.6%)	
Master level diploma	9 (4.7%)	2 (3.7%)	6 (5.6%)	0 (0.0%)	
Graduate level diploma	4 (2.1%)	1 (1.9%)	2 (1.9%)	1 (5.6%)	
Not available	8 (4.2%)	5 (9.3%)	2 (1.9%)	2 (11.1%)	
Unknown	15 (7.9%)	7 (13.0%)	16 15.0%)	4 (22.2%)	
Weight (kg)	88.4±28.4	91.7±27.8	85.6±21.3	84.1±22.7	0.49
Height (cm)	169.1±10.6	171.5±10.2	171.0±10.3	165.2±12.0	0.08
BMI (kg/m^2^)	30.4±8.1	30.8±9.1	29.3±7.2	30.6±6.7	0.63
Obesity	83 (43.4%)	26 (48.1%)	38 (35.2%)	9 (50%)	0.28
Cohort: MN	40 (21.1%)	15 (28.3%)	34 (31.8%)	5 (27.8%)	0.53
MCD	37 (19.5%)	11 (20.8%)	15 (14.0%)	2 (11.1%)	
Other	50 (26.3%)	9 (17.0%)	25 (23.4%)	3 (16.7%)	
FSGS	63 (33.2%)	18 (34.0%)	33 (30.8%)	8 (44.4%)	
Hypertensive Status: Normal	75 (1 (39.3%)	14 (25.9%)	39 (36.1%)	11 (61.1%)	0.62
Elevated BP	84 (44.0%)	26 (48.1%)	37 (34.3%)	6 (33.3%)	
Hypertensive	32 (16.8%)	14 (25.9%)	32 (29.6%)	1 (5.6%)	
SBP (mmHg)	122.8±17.3	129.8±21.7	125.6±22.0	119.9±17.2	0.09
SBP Index	0.79±0.13	0.78±0.15	0.75±0.15	0.79±0.12	0.25
DBP (mmHg)	75.5±11.9	76.8±11.9	76.4±12.1	72.9±10.2	0.38
eGFR (ml/min/1.73m^2^)	69.5±31.0	74.6±31.8	67.4±29.7	55.8±37.6	0.15
UPC ratio (g/g)	3.3±4.5	4.0±5.4	3.7±3.8	4.2±3.9	0.71
Edema present	85 (44.5%)	29 (53.7%)	49 (45.4%)	10 (55.6%)	0.56
Albumin (mg/dl)	3.2± 0.10	2.9±0.9	3.1±1.0	3.0±1.0	0.56
Total Cholesterol (mg/dL)	262.0±111.5	271.3±96.9	259.3±91.8	257.5±93.1	0.91
HDL (mg/dL)	67.1±30.8	62.6±25.9	69.5±30.1	57.8±16.4	0.30
LDL (mg/dL)	156.5±90.2	159.9±83.2	150.5±77.0	152.0±84.7	0.91
Triglycerides (mg/dL)	192.2±128.4	243.9±152.0	196.8±162.3	238.4±156.2	0.08
Steroid use	40 (20.9%)	14 (25.9%)	21 (19.4%)	3 (16.7%)	0.77
CNI use	6 (3.1%)	3 (5.6%)	1 (0.9%)	0 (0.0%)	0.31
RAAS blocker use	109 (57.1%)	31 (57.4%)	56 (51.9%)	11 (61.1%)	0.78
Arterial Hyalinosis	0.35±0.55	0.43±0.86	1.02±1	0.19±0.53	0.11
Arteriosclerosis (%)	0.81±0.9	0.93±0.56	0.23±0.46	0.31±0.46	0.17

BMI – body mass index; MN –membranous nephropathy; MCD- minimal change disease; FSGS- focal segmental glomerulosclerosis; BP – blood pressure; SBP- systolic blood pressure; DBP- diastolic blood pressure; eGFR- estimated glomerular filtration rate; UPC- urine protein:creatinine ratio; HDL – high density lipoprotein cholesterol; LDL – low density lipoprotein cholesterol; CNI - calcineurin inhibitor; RAAS – renin angiotensin aldosterone system

Among the pediatric cohort, the mean age was 9.8 ± 5 years, 57.3% of the participants were male and 39.4% identified as Black race (Table 2). The median duration of follow up was 4.1 (IQR 2.3, 4.7) years. The prevalence of self-reported tobacco exposure at baseline was 81.3% (N = 156) non-smokers, 0.05% (N = 1) active smoker, 1.6% (N = 3) former smokers and 16.7% (N = 32) exclusive passive smokers. When comparing the passive smokers with non-smokers, there were significant differences in the mean age and maternal education level between the two groups.

**Table 2:** Baseline Demographic and Clinical Characteristics of Pediatric NEPTUNE Participants by Tobacco Exposure Groups

N (%) or Mean±SD N = 188^a^	Non-smoker N = 156	Exclusive Passive smoker N = 32	p-value
Age	10.1±4.91	8.0±4.9	0.04
Sex: Male	88 (56.4%)	19 (59.4%)	0.84
Black Race	60 (39.5%)	11 (39.5%)	0.69
Drug use	0 (0.0%)	0 (0.0%)	>0.99
Alcohol use: None	134 (97.8%)	30 (93.8%)	0.05
Daily	0 (0.0%)	1 (3.1%)	
3-4 times a week	0 (0.0%)	0 (0.0%)	
1-2 times a week	1 (0.7%)	0 (0.0%)	
1-2 times a month	2 (1.5%)	0 (0.0%)	
< once a month	0 (0.0%)	1 (3.1%)	
English as primary language	138 (88.5%)	30 (96.8%)	0.21
Maternal Education Level:			0.01
High School diploma	24 (15.4%)	7 (21.9%)	
2-year Associates degree/certificate	42 (26.9%)	4 (12.5%)	
4-year College degree	27 (17.3%)	4 (12.5%)	
Master level diploma	8 (5.1%)	0 (0.0%)	
Graduate level diploma	7 (4.5%)	0 (0.0%)	
Not available	4 (2.6%)	1 (3.1%)	
Unknown	5 (3.2%)	1 (3.1%)	
BMI (kg/m^2^)	22.3±6.7	21.8±7.0	0.3
BMI percentile	76.6±28.2	82.2±25.3	0.16
Obesity	49 (0.3)	17 (53.1)	0.12
Birth weight (g)	4610.0±7316.6	3501.2±260.3	0.27
Cohort: MN	1 (0.6%)	0 (0.0%)	0.76
MCD	80 (51.6%)	19 (59.4%)	
Other	27 (17.4%)	6 (18.8%)	
FSGS	47 (30.3%)	7 (21.9%)	
Blood Pressure Status: Normal	72 (48.6%)	13 (43.3%)	0.87
Elevated BP	14 (9.5%)	3 (10.0%)	
Hypertensive BP	62 (41.9%)	14 (46.7%)	
SBP (mmHg)	111.43±15.403	106.70±18.904	0.12
SBP Index	0.9425±0.11553	0.9418±0.08770	0.79
DBP (mmHg)	76.3±11.4	72.9±10.2	0.04
DBP index	0.9±0.56	0.94±0.18	0.41
eGFR (ml/min/1.73m^2^)	99.2±33.3	121.2±68.4	0.06
UPC ratio (g/g)	3.23±6.2	3.6 ±3.9	0.80
Edema present	55 (36.7%)	16 (50.0%)	0.17
Albumin (mg/dl)	3.2±1.0	2.9±1.2	0.26
Total Cholesterol (mg/dL)	295.3±136.3	316.8±145.7	0.41
HDL (mg/dL)	77.0±28.1	82.2±30.9	0.32
LDL (mg/dL)	179.4±108.4	197.3±115.8	0.4
Triglycerides (mg/dL)	194.4±155.4	186.5±167.7	0.69
Steroid use	90 (46.7%)	19 (59.4%)	>0.99
CNI use	30 (19.2%)	8 (25.0%)	0.67
RAAS blocker use	50 (32.1%)	9 (28.1%)	0.57
Arterial Hyalinosis	0.01±0.07	0.05±0.23	0.36
Arteriosclerosis (%)	0.06±0.21	0.08±0.25	0.69

^a^Total N excludes one current smoker and 3 previous smokers

BMI – body mass index; MN –membranous nephropathy; MCD- minimal change disease; FSGS- focal segmental glomerulosclerosis; BP – blood pressure; SBP- systolic blood pressure; DBP- diastolic blood pressure; eGFR- estimated glomerular filtration rate; UPC- urine protein:creatinine ratio; HDL – high density lipoprotein cholesterol; LDL – low density lipoprotein cholesterol; CNI - calcineurin inhibitor; RAAS – renin angiotensin aldosterone system

### Cardiovascular risk factor outcomes

At baseline, there were no significant differences in BP or lipid measurements among the tobacco exposure groups in the adult and pediatric cohorts (Tables 1 and 2). Over time, there were also no significant differences in self-reported hypertension diagnosis among the adult and pediatric cohorts (Table 3). In longitudinal adjusted regression models, active smoking (reference non-smoking) was significantly associated with greater total cholesterol (β 17.91 95% CI 0.06, 35.76, p = 0.049) among adults. No significant associations were found between active smoking (reference non-smoking) and hypertensive BP status, systolic or diastolic BP, HDL, LDL or triglycerides in adults (Table 4). Among children no significant relationships were found between passive smoking and BP status, systolic/diastolic BP or lipid studies (Table 5).

**Table 3:** Outcomes among NEPTUNE Participants by Tobacco Exposure Groups

	Adults				Pediatric	
N (%) N = 371	**Non-smoker** N = 191	**Active smoker** N = 54	**Former smoker** N = 108	**Exclusive Passive smoker** N = 18	**Non-smoker** N = 156	**Exclusive Passive smoker** N = 32
**Cardiovascular Disease Risk Factors**						
Self-reported Hypertension at Baseline	115 (60.2%)	32 (59.3%)	67 (62%)	13 (72.2%)	34 (21.8%)	10 (31.2%)
Self-reported Hypertension after Baseline	5 (2.6%)	1 (1.9%)	2 (1.9)	0 (0%)	5 (3.2%)	4 (12.5%)
**Cardiovascular Disease Events**						
Coronary Heart Disease at Baseline	13 (6.8%)	5 (9.3%)	5 (4.6%)	0 (0%)	0 (0%)	0 (0%)
Coronary Heart Disease after Baseline	1 (0.5%)	1 (1.9%)	1 (0.93%)	0 (0%)	0 (0%)	0 (0%)
Heart Failure at Baseline	5 (2.6%)	3 (5.6%)	1 (0.93%)	0 (0%)	0 (0%)	0 (0%)
Heart Failure after Baseline	4 (2.1%)	0 (0%)	1 (0.93%)	0 (0%)	1 (0.64%)	0 (0%)
Stroke at Baseline	3 (1.6%)	4 (7.4%)	5 (4.6%)	1 (5.6%)	0 (0%)	0 (0%)
Stroke after Baseline	1 (0.5%)	1 (1.9%)	2 (1.9%)	0 (0%)	0 (0%)	0 (0%)
Peripheral Vascular Disease at Baseline	2 1%)	2 (3%)	2 (1.9%)	0 (0%)	0 (0%)	0 (0%)
Peripheral Vascular Disease after Baseline	2 (1%)	0 (0%)	0 (0%)	0 (0%)	0 (0%)	0 (0%)
**Kidney Disease Outcomes**						
Complete Remission	98 (51.3)	23 (42.6)	59 (54.6)	6 (50%)	111 (71.2%)	23 (71.9%)
Chronic Kidney Disease Progression*	39 (20.4)	13 (24.1)	26 (24.1)	4 (22.2%)	31 (19.9%)	4 (12.5%)
Kidney Failure	16 (8.4)	4 (7.4)	8 (7.4)	2 (11.1%)	1 (0.64%)	0 (0%)
**Co-Morbid Diagnoses**						
Cancer at Baseline	15 (7.9%)	5 (9.3%)	5 (4.6%)	1 (5.6%)	0 (0%)	0 (0%)
Cancer after Baseline	4 (2.1%)	2 (3.7%)	3 (2.8%)	1 (5.6%)	1 (0.64%)	0 (0%)
Thromboemboilic Events at Baseline**	6 (3.1%)	1 (1.9%)	10 (9.3%)	0 (0%)	4 (2.6%)	0 (0%)
Thromboembolic Events after Baseline	6 (3.1%)	1 (1.9%)	4 (3.7%)	0 (0%)	1 (0.64%)	0 (0%)

*For children, chi-square test p = 0.03, **For adults, p = 0.04. All other tests p >0.05

**Table 4:** Association between Tobacco Exposure and Cardiovascular Risk Factors and Kidney-related Outcomes in Adjusted Regression Models (reference group: non-smokers)^a^ in Adult NEPTUNE Participants

	Active Smokers			Exclusive Passive Smokers	
	**OR (95% CI)**		p value	**OR (95% CI)**	p value
Hypertensive BP	0.51 (0.22, 1.16)		0.11	1.26 (0.84, 1.90)	0.26
Dyslipidemia	1.29 (0.78, 2.13)		0.32	Failed to Converge	
Complete remission	0.86 (0.70, 1.05)		0.13	1.00 (0.79, 1.27)	0.98
	**β (95% CI)**		p value	**β (95% CI)**	p value
Systolic BP (mmHg)	1.26 (-1.21, 3.72)		0.32	0.363 (-1.88, 2.61)	0.75
Diastolic BP (mmHg)	0.43 (-2.05, 2.91)		0.73	0.01 (-2.00, 2.01)	0.99
HDL (mg/dl)	0.00 (-3.75, 3.75)		1	1.34 (-6.04, 8.71)	0.72
LDL (mg/dl)	7.12 (-4.01, 18.24)		0.21	-6.79 (-29.78, 16.21)	0.56
Triglycerides (mg/dl)	3.41 (-31.02, 37.84)		0.58	-21.86 (-59.13, 15.40)	0.25
Total Cholesterol (mg/dl)	17.91 (0.06, 35.76)		0.049	-38.85 (-87.21, 9.51)	0.12
UPCr (g/g)	0.39 (-0.09, 0.87)		0.12	1.23 (0.13, 2.33)	0.03
eGFR (ml/min/1.73m2)	3.06 (-2.96, 9.07)		0.32	4.13 (-2.52, 10.78)	0.22
	** h (95% CI)**	p value		**HR (95% CI)**	p value
CKD progression	1.24 (0.91, 1.68)	0.17		0.71 (0.48, 1.04)	0.08
Kidney failure	0.82 (0.51, 1.32)	0.41		-	-

^a^ Models were adjusted for age, sex, weight status, race, glomerular diagnosis, UPC, log eGFR and steroids. Exceptions: UPC model did not include UPC. eGFR model did not include eGFR. Kidney Failure adjusted only for age and sex due to small number of events in adults. Kidney failure not performed in children due to small number of events. CKD progression in children adjusted for age and sex due to small number of events

**Table 5:** Association between Tobacco Exposure and Cardiovascular Risk Factors and Kidney-related Outcomes in Adjusted Regression Models (reference group: non-smokers)^a^ in Pediatric NEPTUNE Participants

	Passive Smokers	
	**OR (95% CI)**	p value
Hypertensive BP	1.26 (0.84, 1.90)	0.26
Dyslipidemia	Failed to Converge	
Complete remission	1.00 (0.79, 1.27)	0.98
	**β (95% CI)**	p value
Systolic BP (mmHg)	0.363 (-1.88, 2.61)	0.75
Diastolic BP (mmHg)	0.01 (-2.00, 2.01)	0.99
HDL (mg/dl)	1.34 (-6.04, 8.71)	0.72
LDL (mg/dl)	-6.79 (-29.78, 16.21)	0.56
Triglycerides (mg/dl)	-21.86 (-59.13, 15.40)	0.25
Total Cholesterol (mg/dl)	-38.85 (-87.21, 9.51)	0.12
UPCr (g/g)	1.23 (0.13, 2.33)	0.03
eGFR (ml/min/1.73m2)	4.13 (-2.52, 10.78)	0.22
	** h (95% CI)**	p value
CKD progression	0.71 (0.48, 1.04)	0.08

^a^ Models were adjusted for age, sex, weight status, race, glomerular diagnosis, UPC, log eGFR and steroids. Exceptions: UPC model did not include UPC. eGFR model did not include eGFR. Kidney Failure adjusted only for age and sex due to small number of events in adults. Kidney failure not performed in children due to small number of events. CKD progression in children adjusted for age and sex due to small number of events

### Cardiovascular disease events and co-morbid conditions

Cardiovascular disease events, cancer diagnosis and thromboembolic events reported at baseline and during follow-up by tobacco exposure are presented in Table 3. Among adults, there was a significantly higher frequency of thromboembolic events (9.3%) reported at the baseline visit in former smokers compared to the other tobacco exposure groups. There were no other significant differences for other disease conditions among the adult and pediatric cohorts.

### Kidney disease outcomes

At baseline, there were no significant differences in UPC or eGFR among the tobacco exposure groups in the adult and pediatric cohorts (Tables 1 and 2). Over time, there were no significant differences in the frequency of complete remission or kidney failure among adults and children. For children, there was a significantly higher frequency of CKD progression in non-smokers compared to those exposed to passive smoke (Table 3). In longitudinal adjusted regression models, no significant associations were found between active smoking (reference non-smoking) and UPC, eGFR, nephrotic syndrome remission, CKD progression, or development of kidney failure in adults (Table 4). In children, passive smoking (reference non-smoking) was significantly associated with greater UPC over time (β 1.23 95% CI 0.13, 2.33, p = 0.03). No relationships between passive smoking and remission of nephrotic syndrome or CKD progression were found (Table 5).

### Pathology

There were no significant differences in kidney tissue arterial hyalinosis score or arteriosclerosis percentage among the tobacco exposure groups in the adults and pediatric cohorts (Tables 1 and 2).

### Serum cotinine

Serum cotinine was measured in a sub-population of the cohort, which included 123 adults and 55 children. There were no significant differences in baseline demographic and clinical characteristics between the cotinine sub-cohort and those without cotinine measurements (data not shown). Classification of tobacco exposure by cotinine criteria included 95 (53.4%) non-smokers, 30 (16.9%) active smokers and 53 (29.8%) passive smokers.

Compared to the cotinine tobacco exposure classification, the sensitivity of self-reported active smoking was 75.9% and the specificity was 97.7%. The sensitivity and specificity for self-reported passive smoking were low compared to cotinine measures, 17.3% and 11.6%, respectively. Of those classified as non-smokers by cotinine, there were 10 who self-reported passive smoke exposure. Of the active smokers by cotinine, there were two who reported non-smoking status and one who reported passive smoke exposure. Of the passive smokers by cotinine, there were two reports of active smoking and 33 reports of non-smoking exposure.

There was a positive correlation between serum cotinine and baseline eGFR (r = 0.17, p = 0.03). There were no significant correlations between serum cotinine and baseline UPC, BP or lipids.

## Discussion

In NEPTUNE, the self-reported prevalence of active smoking was 14.6% in adults and the prevalence of exclusive passive smoking was 4.9% in adults and 16.7% in children. Active smoking was significantly associated with greater total cholesterol over time in adults while passive smoke exposure was associated with greater proteinuria over time in children. The sensitivity of self-reported smoking exposure status compared to serum cotinine levels was modest for active smoking and poor for passive smoking. Higher serum cotinine, measured in a sub-cohort at baseline, correlated with a higher eGFR.

The prevalence of active smoking in adults from NEPTUNE is similar to the reported 14% of adults in the United States who smoke cigarettes [1]. Smoke exposure in children was lower than expected from previous studies in the general pediatric population; these general pediatric studies describe a larger percentage of children who are active smokers or who are exposed to second-hand smoke. One study of 182 adolescents showed that 52% lived with an adult who smokes and 24% had smoked at some point in their lives [5]. On the other hand, similar to our findings, a Chronic Kidney Disease in Children (CKiD) study found that 2% of children ages 13–18 years were active smokers and 20% had passive smoke exposure [26]. Similar to other studies, the concordance between serum cotinine and self-report of tobacco exposure was not strong, suggesting that smoke exposure was likely under-reported [27].

Active and passive smoke exposure are associated with increased CVD risk in adults [3]. Smoking is a risk factor for coronary heart disease and progression of atherosclerotis [3, 28, 29]. Second-hand smoking has been shown to increase the risk of CVD by as much as 25–30% in pooled estimates from a meta-analyses [30] and there is supportive evidence of a causal association between second-hand smoke exposure and CVD mortality in nonsmokers [31]. Second-hand smoke is also an independent risk factor for the development of CVD in children. Children with second-hand smoke exposure were found to have higher blood pressure, blood pressure variability and endothelial dysfunction [4, 32].

Active smoking was associated with greater total cholesterol in adults in this present study suggesting an increased cardiovascular risk profile related to tobacco exposure in adults with proteinuric glomerulopathies. These results are in agreement with previous studies that have shown that smoking leads to an increase in lipid levels [33–37]. The mechanisms of this phenomenon have been described by Valkonen and Kuusi: tobacco exposure results in loss of antioxidant defense, a reduction in ascorbic acid, and an accumulation of LDL in cultured human macrophages [37]. These mechanisms lead to a decrease in LDL oxidation, increased peroxidation, and an overall increase in lipid levels, thereby increasing the risk of atherosclerosis [38–42].

Tobacco exposure is also a known risk factor for progression of kidney disease and proteinuria [5–7]. In adults, smoking is an independent risk factor for transplant nephropathy [43] and progression of CKD to kidney failure [44]. In a systematic review of 17 studies, there was a significant association between smoking and incident CKD [45]. A national study of 13,000 adults found that active smokers were 1.85 times more likely to have albuminuria than nonsmokers. Of nonsmokers, studies also support the association of passive smoke exposure with albuminuria and CKD in adults [6, 46, 47]. Contrary to expectations, our study did not find any associations between tobacco exposure and kidney disease outcomes in adults with proteinuric glomerulopathies. The finding that passive smoking was associated with proteinuria in children with proteinuric glomerulopathies is notable, as this suggests that tobacco exposure may affect kidney outcomes early in life. In CKiD, passive smoke exposure was associated with a 2.64 odds of nephrotic-range proteinuria compared to unexposed children [5]. Passive smoke exposure has also been associated with a decline in eGFR in adolescents [48]. The current hypothesis is that smoking triggers a sympathetic response causing intra-renal vasoconstriction. This leads to increased glomerular pressure and kidney hyperperfusion leading to structural alterations of the glomeruli and eventually hyperfiltration [49–51]. Glomerular hyperfiltration often results in an increase in proteinuria [49–51].

**Fig. 1:**
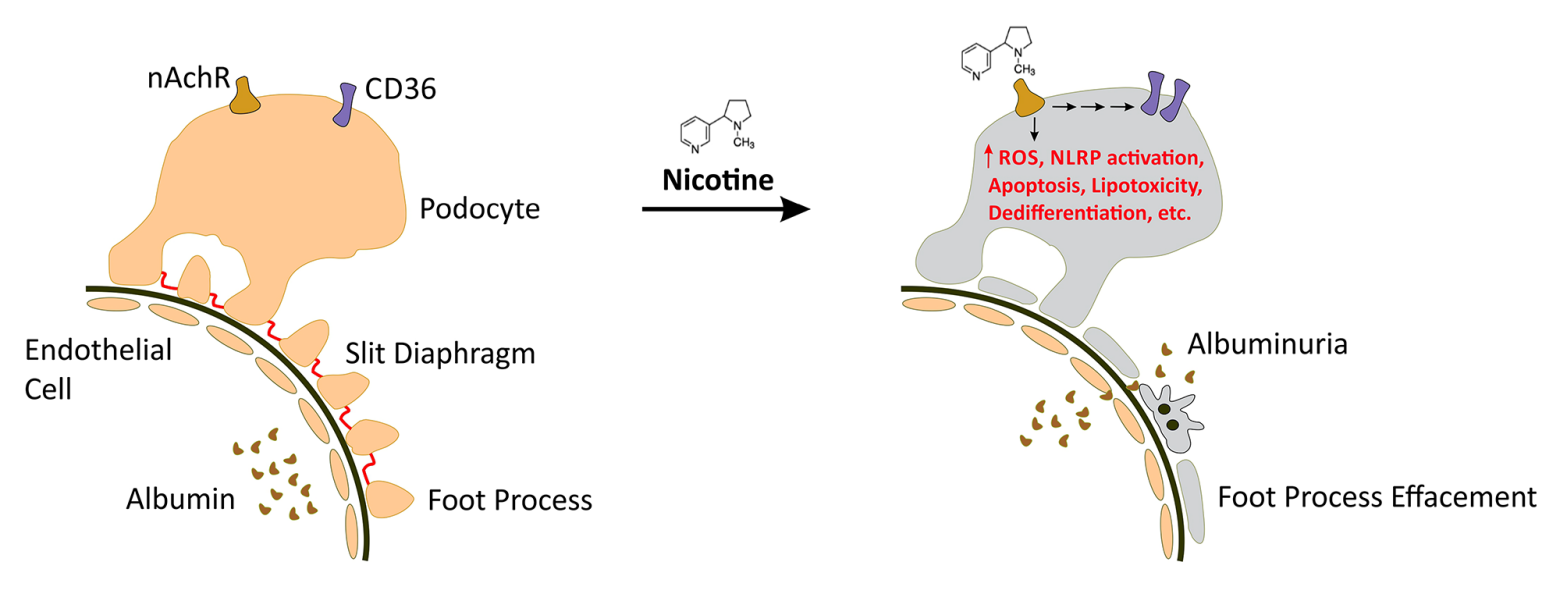
**Known Mechanisms of Nicotine-induced Podoyte Injury**. Mature podocytes express the nicotinic acetylcholine receptor (nAchR). Exposure to nicotine activates signaling through the nAchR which drives reactive oxygen species generation, proinflammatory cytokine expression, activation of the NLRP inflammasome, downregulation of podocyte maturity marker expression and apoptosis. Podocyte CD36 expression is also upregulated with nicotine exposure which may promote liptoxicity through the enhanced uptake of oxidized LDL (oxLDL).

There is considerable evidence of the direct cytotoxic effects of nicotine and its metabolites on podocyte function. Zarzecki et al. demonstrated that Sprague-Dawley rats exposed *in utero* to cigarette smoke extract, exhibited significantly reduced glomerular volume and podocyte number suggesting that prenatal exposure to nicotine and its metabolites may negatively impact glomerular development [8]. Subsequently, Singh et al. demonstrated that nicotine exposure induces activation of the NLRP inflammasome, reduced expression of podocyte maturity markers (i.e. podocin and nephrin), upregulation of caspase 1 and IL-1β expression and increased cellular permeability in vitro (Fig. 1) [11]. Jaimes et al. recently showed that nicotine binds directly to podocytes via the nicotinic acetylcholine receptor (nAchR) to induce reactive oxygen species generation and COX-2 expression, increased CD36-mediated oxLDL uptake, reduced podocyte maturity marker expression (i.e. synaptopodin), fibronectin expression, apoptosis and glomerular injury (Fig. 1) [9]. Taken together, these studies demonstrate the direct cytotoxicity of nicotine and its metabolites on podocytes, which may contribute to proteinuria via disruption of glomerular filtration barrier integrity.

We found a weak, but significant, positive correlation between serum cotinine and baseline eGFR. This is consistent with previous studies that showed that increased eGFR is associated with smoking [50, 51, 52, 53]. On the other hand, a few studies found the opposite effect of smoking on eGFR [48, 54]. Intriguingly, Pinto-Sietsma et al. found both a significant increase and a significant decrease in eGFR to be correlated with smoking among 7476 participants [51]. They suggest that decreased eGFR may be due to an initial decrease in renal plasma flow that is caused by smoking before glomeruli are damaged with subsequent compensatory glomerular hyperfiltration driving the increase in eGFR [55, 56]. Cystatin C measurements provide another perspective on the effects of smoking on eGFR. It has been shown that cystatin C-based calculations of eGFR predict cardiovascular outcomes, end stage renal disease, and death better than creatinine-based eGFR calculations [57]. Additionally, one study showed that active smoking was associated with a higher creatinine-based eGFR, but was not associated with a change in cystatin C-based eGFR [58]. Further investigations of the relationship between serum cotinine levels and creatinine-based eGFR compared to cystatin C-based eGFR may help clarify conflicting study results.

This study has several limitations. As mentioned previously, self-reported tobacco exposure is generally under-reported [27], which may attenuate relationships between tobacco exposure and cardiovascular risk factor and kidney disease outcomes. Another limitation is that the NEPTUNE protocol did not specifically include questions about vaping or electronic cigarette use, which may have led to an under-estimation of the true tobacco exposure, especially in the adolescent population. Given the observational nature of the study, there are possible confounding factors (e.g. diet, socioeconomic factors, comorbidities, genetics) that were not captured and may have had an effect on the outcomes of interest. Also, the duration of follow-up may not have been long enough to capture some of the outcomes of interest. Lastly, results may not be generalizable to other practice settings since all NEPTUNE study sites are academic centers.

In conclusion, tobacco exposure is prevalent among adults and children with proteinuric glomerulopathies and is associated with higher cardiovascular risk profiles and worse kidney disease outcomes. Active smoking was significantly associated with greater total cholesterol in adults and passive smoking was significantly associated with proteinuria in children. Therefore, preventive strategies to reduce smoke exposure, including counseling of caregivers of children with glomerulopathies who might be sources of passive tobacco exposure, is important to mitigate risks of cardiovascular and kidney complications in patients with proteinuric glomerulopathies.

## Electronic supplementary material

Below is the link to the electronic supplementary material.

Supplementary Material 1

## Data Availability

The datasets generated and/or analyzed during the current study are available in the NIH repository.

## References

[1] CDC. Current Cigarette Smoking Among Adults in the United States. Centers for Disease Control and Prevention. Published December 15, 2020. Accessed December 30., 2021. https://www.cdc.gov/tobacco/data_statistics/fact_sheets/adult_data/cig_smoking/index.htm

[2] Products - Data Briefs. - Number 396 - February 2021. doi:10.15620/cdc:101197

[3] Office on Smoking and Health (US). The Health Consequences of Involuntary Exposure to Tobacco Smoke: A Report of the Surgeon General. Centers for Disease Control and Prevention (US). ; 2006. Accessed December 30, 2021. http://www.ncbi.nlm.nih.gov/books/NBK44324/20669524

[4] Pijanowska M, Zajaczkowska M (2004). [Passive smoking and patterns of 24-hour ambulatory blood pressure in healthy children]. Pol Merkur Lekarski.

[5] Omoloja A, Chand D, Greenbaum L (2011). Cigarette smoking and second-hand smoking exposure in adolescents with chronic kidney disease: a study from the Midwest Pediatric Nephrology Consortium. Nephrol Dialysis Transplantation.

[6] Hogan SL, Hogan SL, Vupputuri S (2007). Association of cigarette smoking with Albuminuria in the United States: the Third National Health and Nutrition Examination Survey. Ren Fail.

[7] Jaimes EA, Tian RX, Raij L (2007). Nicotine: the link between cigarette smoking and the progression of renal injury?. Am J Physiol Heart Circ Physiol.

[8] Zarzecki M, Adamczak M, Wystrychowski A, Gross ML, Ritz E, Więcek A (2012). Exposure of pregnant rats to cigarette-smoke condensate causes glomerular abnormalities in offspring. KBR.

[9] Jaimes EA, Zhou MS, Siddiqui M (2021). Nicotine, smoking, podocytes, and diabetic nephropathy. Am J Physiol Renal Physiol.

[10] Wakino S, Hasegawa K, Itoh H (2015). Sirtuin and metabolic kidney disease. Kidney Int.

[11] Singh GB, Kshirasagar N, Patibandla S, Puchchakayala G, Koka S, Boini KM (2019). Nicotine instigates podocyte injury via NLRP3 inflammasomes activation. Aging.

[12] Lan X, Lederman R, Eng JM (2016). Nicotine induces Podocyte apoptosis through increasing oxidative stress. PLoS ONE.

[13] IBRAHIM ZS, ALKAFAFY ME, AHMED MM, SOLIMAN MM (2016). Renoprotective effect of curcumin against the combined oxidative stress of diabetes and nicotine in rats. Mol Med Rep.

[14] Roth KS, Amaker BH, Chan JCM (2002). Nephrotic syndrome: pathogenesis and management. Pediatr Rev.

[15] Kodner C (2016). Diagnosis and management of nephrotic syndrome in adults. AFP.

[16] Kolb A, Gallacher PJ, Campbell J (2021). A National Registry Study of patient and renal survival in adult nephrotic syndrome. Kidney Int Rep.

[17] Gadegbeku CA, Gipson DS, Holzman LB (2013). Design of the nephrotic syndrome Study Network (NEPTUNE) to evaluate primary glomerular nephropathy by a multidisciplinary approach. Kidney Int.

[18] Benowitz NL, Hukkanen J, Jacob P. Nicotine Chemistry, metabolism, kinetics and biomarkers. Handb Exp Pharmacol. 2009;19229–60. 10.1007/978-3-540-69248-5_2. 10.1007/978-3-540-69248-5_2 PMC2953858 19184645

[19] Flynn JT, Kaelber DC, Baker-Smith CM (2017). Clinical practice Guideline for Screening and Management of High Blood pressure in children and adolescents. Pediatrics.

[20] Fuchs FD, Whelton PK (2020). High blood pressure and Cardiovascular Disease. Hypertension.

[21] 2017 Guideline for High Blood Pressure in Adults. American College of Cardiology. Accessed December 30., 2021. https://www.acc.org/latest-in-cardiology/ten-points-to-remember/2017/11/09/11/41/http%3a%2f%2fwww.acc.org%2flatest-in-cardiology%2ften-points-to-remember%2f2017%2f11%2f09%2f11%2f41%2f2017-guideline-for-high-blood-pressure-in-adults

[22] Expert Panel on Integrated Guidelines for Cardiovascular Health and Risk Reduction in Children and Adolescents (2011). Summary Rep Pediatr.

[23] Levey AS, Stevens LA, Schmid CH (2009). A New equation to Estimate glomerular filtration rate. Ann Intern Med.

[24] Schwartz GJ, Muñoz A, Schneider MF (2009). New equations to estimate GFR in children with CKD. J Am Soc Nephrol.

[25] CDC. BMI for Children and Teens. Centers for Disease Control and Prevention. Published December 3, 2021. Accessed January 9., 2022. https://www.cdc.gov/obesity/childhood/defining.html

[26] Omoloja A, Stolfi A, Chand D (2014). Tobacco exposure in children and adolescents with chronic kidney disease: parental behavior and knowledge. A study from the Midwest Pediatric Nephrology Consortium. Clin Nephrol.

[27] Connor Gorber S, Schofield-Hurwitz S, Hardt J, Levasseur G, Tremblay M (2009). The accuracy of self-reported smoking: a systematic review of the relationship between self-reported and cotinine-assessed smoking status. Nicotine Tob Res.

[28] Strong JP, Richards ML (1976). Cigarette smoking and atherosclerosis in autopsied men. Atherosclerosis.

[29] Zieske AW, McMahan CA, McGill HC (2005). Smoking is associated with advanced coronary atherosclerosis in youth. Atherosclerosis.

[30] Women and Smoking: A Report of the Surgeon General.

[31] Health effects of exposure to environmental tobacco smoke (1997). California Environmental Protection Agency. Tob Control.

[32] Kallio K, Jokinen E, Raitakari OT (2007). Tobacco smoke exposure is Associated with attenuated endothelial function in 11-Year-old healthy children. Circulation.

[33] Hallit S, Zoghbi M, Hallit R (2017). Effect of exclusive cigarette smoking and in combination with waterpipe smoking on lipoproteins. J Epidemiol Glob Health.

[34] Venkatesan A, Hemalatha A, Bobby Z, Selvaraj N, Sathiyapriya V (2006). Effect of smoking on lipid profile and lipid peroxidation in normal subjects. Indian J Physiol Pharmacol.

[35] Batic-Mujanovic O, Beganlic A, Salihefendic N, Pranjic N, Kusljugic Z (2008). Influence of smoking on serum lipid and lipoprotein levels among family medicine patients. Med Arh.

[36] Rao Ch S, Subash YE (2013). The effect of chronic tobacco smoking and chewing on the lipid profile. J Clin Diagn Res.

[37] Valkonen M, Kuusi T, Lipoprotein L, density, Valkonen M. Passive smoking induces atherogenic changes in low-density lipoprotein.Circulation97. Published online 2012. 10.1161/01.cir.97.20.2012 9610530

[38] Church DF, Pryor WA (1985). Free-radical chemistry of cigarette smoke and its toxicological implications. Environ Health Perspect.

[39] Roberts LJ, Morrow JD (2000). Measurement of F(2)-isoprostanes as an index of oxidative stress in vivo. Free Radic Biol Med.

[40] Lawson JA, Rokach J, FitzGerald GA (1999). Isoprostanes: formation, analysis and use as indices of lipid peroxidation in vivo. J Biol Chem.

[41] Maiorino M, Zamburlini A, Roveri A, Ursini F (1993). Prooxidant role of vitamin E in copper induced lipid peroxidation. FEBS Lett.

[42] Roberts K, Rezai A a., Pinkerton K e., Rutledge J. c. Effect of Environmental Tobacco Smoke on LDL Accumulation in the Artery Wall. *Circulation*. 1996;94(9):2248–2253. doi:10.1161/01.CIR.94.9.2248 10.1161/01.cir.94.9.2248 8901679

[43] Zitt N, Kollerits B, Neyer U (2007). Cigarette smoking and chronic allograft nephropathy. Nephrol Dialysis Transplantation.

[44] Orth SR, Hallan SI (2008). Smoking: a risk factor for progression of chronic kidney disease and for cardiovascular morbidity and mortality in renal patients–absence of evidence or evidence of absence?. Clin J Am Soc Nephrol.

[45] Jones-Burton C, Seliger SL, Scherer RW (2007). Cigarette smoking and incident chronic kidney disease: a systematic review. Am J Nephrol.

[46] Dülger H, Dönder A, Sekeroğlu MR, Erkoç R, Ozbay B (2011). Investigation of the relationship between serum levels of cotinine and the renal function in active and passive smokers. Ren Fail.

[47] Yamagata K, Ishida K, Sairenchi T (2007). Risk factors for chronic kidney disease in a community-based population: a 10-year follow-up study. Kidney Int.

[48] García-Esquinas E, Loeffler LF, Weaver VM, Fadrowski JJ, Navas-Acien A (2013). Kidney function and Tobacco smoke exposure in US adolescents. Pediatrics.

[49] Halimi JM, Giraudeau B, Vol S (2000). Effects of current smoking and smoking discontinuation on renal function and proteinuria in the general population. Kidney Int.

[50] Baggio B, Budakovic A, Dalla Vestra M, Saller A, Bruseghin M, Fioretto P (2002). Effects of cigarette smoking on glomerular structure and function in type 2 diabetic patients. J Am Soc Nephrol.

[51] Pinto-Sietsma SJ, Mulder J, Janssen WM, Hillege HL, de Zeeuw D, de Jong PE (2000). Smoking is related to albuminuria and abnormal renal function in nondiabetic persons. Ann Intern Med.

[52] Vargas F, Romecín P, García-Guillén AI (2018). Flavonoids in kidney health and disease. Front Physiol.

[53] Miyatake N, Moriyasu H, Sakano N, Tada S, Suzue T, Hirao T (2010). Influence of cigarette smoking on estimated glomerular filtration rate (eGFR) in japanese male workers. Acta Med Okayama.

[54] Orth SR, Viedt C, Ritz E (2001). Adverse Effects of Smoking in the renal patient. Tohoku J Exp Med.

[55] Gambaro G, Verlato F, Budakovic A (1998). Renal impairment in chronic cigarette smokers. J Am Soc Nephrol.

[56] Ritz E, Benck U, Franek E, Keller C, Seyfarth M, Clorius J (1998). Effects of smoking on renal hemodynamics in healthy volunteers and in patients with glomerular disease. J Am Soc Nephrol.

[57] Shlipak MG (2013). Cystatin C versus creatinine in determining risk based on kidney function. N Engl J Med.

[58] Mickelsson M (2021). Smoking tobacco is associated with renal hyperfiltration. Scand J Clin Lab Invest.

