# Supplementary material for: Tobacco exposure in adults and children with proteinuric glomerulopathies: a NEPTUNE cohort study
Source: BMC Nephrol. 2023 Feb 9;24:30. doi: 10.1186/s12882-023-03073-w (PMC9912673; doi:10.1186/s12882-023-03073-w)
Supplement: Supplementary file 1 — Supplementary Material 1 [file 12882_2023_3073_MOESM1_ESM.docx]

**Members of the Nephrotic Syndrome Study Network (NEPTUNE)**

NEPTUNE Enrolling Centers

*Cleveland Clinic, Cleveland, OH*: K Dell^*^, J Sedor^**^, M Schachere^#^, J Negrey^#^

*Children’s Hospital, Los Angeles, CA*: K Lemley^*^, J Scott^#^

*Children’s Mercy Hospital, Kansas City, MO*: T Srivastava^*^, S Morrison^#^

*Cohen Children’s Hospital, New Hyde Park, NY:* C Sethna^*^, M Pfaiff ^#^

*Columbia University, New York, NY:* P Canetta^*^, A Pradhan^#^

*Emory University, Atlanta, GA:* L Greenbaum^*^, C Wang**, E Yun^#^

*Harbor-University of California Los Angeles Medical Center:* S Adler^*^, J LaPage^#^

*John H. Stroger Jr. Hospital of Cook County, Chicago, IL:* A Athavale^*^, M Itteera

*Johns Hopkins Medicine, Baltimore, MD:* M Atkinson^*^, T Dell^#^

*Mayo Clinic, Rochester, MN:* F Fervenza^*^, M Hogan**, J Lieske^*^, G Hill^#^

*Montefiore Medical Center, Bronx, NY:* F Kaskel^*^, M Ross^*^, P Flynn^#^

*NIDDK Intramural, Bethesda MD:* J Kopp^*^

*New York University Medical Center, New York, NY:* L Malaga-Dieguez^*^, O Zhdanova**, F Modersitzki^#^, L Pehrson^#^

*Stanford University, Stanford, CA:* R Lafayette^*^, B Yeung^#^

*Temple University, Philadelphia, PA:* I Lee^*^, S Quinn-Boyle^#^

*University Health Network Toronto:* H Reich *, M Hladunewich**, P Ling^#^, M Romano^#^

*University of Miami, Miami, FL:* A Fornoni^*^, C Bidot^#^

*University of Michigan, Ann Arbor, MI:* M Kretzler^*^, D Gipson*, A Williams^#^, C Klida^#^

*University of North Carolina, Chapel Hill, NC:* V Derebail^*^, K Gibson^*^, A Froment^#^, F Ochoa-Toro^#^

*University of Pennsylvania, Philadelphia, PA:* L Holzman^*^, K Meyers**, K Kallem^#^, A Swenson^#^

*University of Texas Southwestern, Dallas, TX:* K Sambandam^*^, K Aleman^#^, M Rogers^#^

*University of Washington, Seattle, WA:* A Jefferson^*^, S Hingorani**, K Tuttle**^§^, L Manahan ^#^, E Pao^#^, A Cooper^#§^

*Wake Forest University Baptist Health, Winston-Salem, NC:* JJ Lin*, Stefanie Baker^#^

*Data Analysis and Coordinating Center*: M Kretzler*, L Barisoni**, C Gadegbeku**, B Gillespie**, D Gipson**, L Holzman**, L Mariani**, M Sampson**, J Sedor**, J Zee**, G Alter, H Desmond, S Eddy, D Fermin, M Larkina, S Li, S Li, CC Lienczewski, T Mainieri, R Scherr, A Smith, A Szymanski, A Williams.

*Digital Pathology Committee*: Carmen Avila-Casado (University Health Network, Toronto), Serena Bagnasco (Johns Hopkins University), Joseph Gaut (Washington University in St Louis), Stephen Hewitt (National Cancer Institute), Jeff Hodgin (University of Michigan), Kevin Lemley (Children’s Hospital of Los Angeles), Laura Mariani (University of Michigan), Matthew Palmer (University of Pennsylvania), Avi Rosenberg (Johns Hopkins University), Virginie Royal (University of Montreal), David Thomas (University of Miami), Jarcy Zee (University of Pennsylvania) Co-Chairs: Laura Barisoni (Duke University) and Cynthia Nast (Cedar Sinai).

*Principal Investigator; **Co-investigator^; #^Study Coordinator

^§^Providence Medical Research Center, Spokane, WA
